# Supplementary material for: An Evaluation of the In Vitro Roles and Mechanisms of Silibinin in Reducing Pyrazinamide- and Isoniazid-Induced Hepatocellular Damage
Source: Int J Mol Sci. 2020 May 25;21(10):3714. doi: 10.3390/ijms21103714 (PMC7279482; doi:10.3390/ijms21103714)
Supplement: Supplementary file 1 [file ijms-21-03714-s001.zip › Supplementary Material/Highlights.docx]

**Highlights:**

- Silibinin preserved cell viability when co-administered with isoniazid
- Silibinin reduced oxidative damage induced by isoniazid and pyrazinamide
- Silibinin maintained mitochondria membrane potential, reducing apoptosis
- Silibinin activated the Nrf2-ARE pathway
