# Supplementary material for: An Evaluation of the In Vitro Roles and Mechanisms of Silibinin in Reducing Pyrazinamide- and Isoniazid-Induced Hepatocellular Damage
Source: Int J Mol Sci. 2020 May 25;21(10):3714. doi: 10.3390/ijms21103714 (PMC7279482; doi:10.3390/ijms21103714)
Supplement: Supplementary file 1 [file ijms-21-03714-s001.zip › Supplementary Material/Supp Figure 1.pdf]

(A) Effect of silibinin on LO-2 viability over 72h

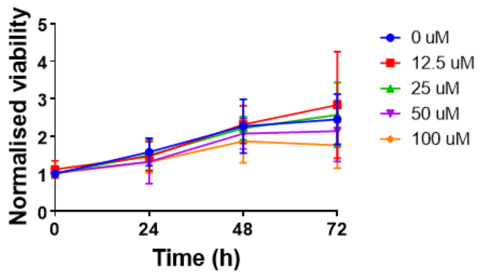

(B) Effect of silibinin on LO-2 viability at 72 h

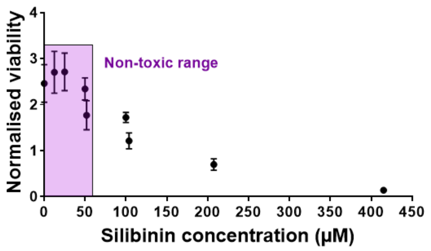

Figure S1 (color, 2-column). Silibinin did not affect LO2 viability at concentrations up to 50 μM over 72 h. (A) Silibinin at concentrations up to 50 μM over 72 h did not hinder LO2 growth. **(B)** After 72 h had passed, silibinin at concentrations up to 50 μM did not hinder LO2 growth, and silibinin above concentrations of 200 μM significantly reduced LO2 viability. Data represent mean ± S.E.M. of at least two replicates.
