# Supplementary material for: An Evaluation of the In Vitro Roles and Mechanisms of Silibinin in Reducing Pyrazinamide- and Isoniazid-Induced Hepatocellular Damage
Source: Int J Mol Sci. 2020 May 25;21(10):3714. doi: 10.3390/ijms21103714 (PMC7279482; doi:10.3390/ijms21103714)
Supplement: Supplementary file 1 [file ijms-21-03714-s001.zip › Supplementary Material/Supp Figure 2.pdf]

(A) 24 h IC_{50} curve of PZA

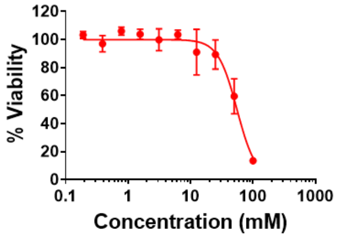

(B) 24 h IC_{50} curve of INH

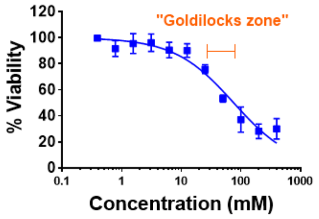

Figure S2 (color, 2-column). PZA and INH reduced LO2 viability when administered over 24 h. (A) The mean IC_{50} of PZA was 60 mM. **(B)** The mean IC_{50} of INH was 73 mM. The “Goldilocks zone” (orange) contained the concentrations of INH that induced toxicity to approximately 50% viability. The more representative IC_{50} curve of two biological replicates is shown for PZA and INH respectively. Data represent mean ± S.D. of six technical replicates.
