# Supplementary material for: An Evaluation of the In Vitro Roles and Mechanisms of Silibinin in Reducing Pyrazinamide- and Isoniazid-Induced Hepatocellular Damage
Source: Int J Mol Sci. 2020 May 25;21(10):3714. doi: 10.3390/ijms21103714 (PMC7279482; doi:10.3390/ijms21103714)
Supplement: Supplementary file 1 [file ijms-21-03714-s001.zip › Supplementary Material/Supp Figure 3.pdf]

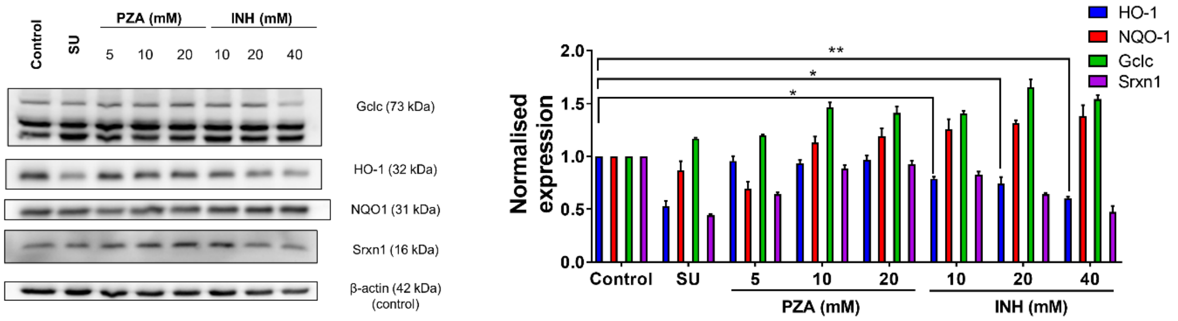

Figure S3 (color, 2-column). INH, but not PZA, significantly suppressed HO-1 expression in LO2. INH suppressed HO-1 expression at 10 mM (one-way ANOVA, $p = 0.0275$), 20 mM (one-way ANOVA, $p = 0.0133$), and 40 mM (one-way ANOVA, $p = 0.0028$); but not Gclc, NQO1, and Srxn1 expression in LO2 *in vitro*. PZA did not have any effect on HO-1, Gclc, NQO1, and Srxn1 at the concentrations tested. Positive control was treated with 10 μM SU for 24 h. Data represent mean ± S.E.M. of two replicates. * $p < 0.05$, ** $p < 0.01$ vs control.
