# Supplementary material for: An Evaluation of the In Vitro Roles and Mechanisms of Silibinin in Reducing Pyrazinamide- and Isoniazid-Induced Hepatocellular Damage
Source: Int J Mol Sci. 2020 May 25;21(10):3714. doi: 10.3390/ijms21103714 (PMC7279482; doi:10.3390/ijms21103714)
Supplement: Supplementary file 1 [file ijms-21-03714-s001.zip › Supplementary Material/Supp Figure 4.pdf]

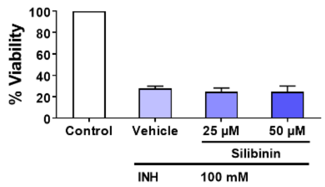

Figure S4 (color, 2-column). Silibinin was not useful as a rescue adjuvant in TAMH. The co-administration of silibinin at 25 and 50 µM did not preserve TAMH function when hepatotoxicity was induced using 100 mM INH. Data represent mean ± S.E.M. of three replicates.
